# Bench to bedside: is rapamycin headed for the docTOR?

**DOI:** 10.1007/s11357-026-02306-8

**Published:** 2026-05-13

**Authors:** Dudley W. Lamming

**Affiliations:** 1 Department of Medicine, University of Wisconsin-Madison, 1685 Highland Ave, MFCB Rm 4147, Madison, WI 53705 USA; 2 Wisconsin Nathan Shock Center of Excellence in the Basic Biology of Aging, Madison, WI 53705 USA

**Keywords:** Rapamycin, Calorie restriction, Malnutrition

## Abstract

Almost a century ago, calorie restriction (CR) was identified as a robust intervention for extending lifespan and healthspan, a discovery that captured the imagination of both scientists and the public. If the powerful mechanisms engaged by CR can be uncovered and harnessed through a pill, humans might be able to live longer and healthier lives. Here, we will discuss the evolution of rapamycin, an inhibitor of the mTOR (mechanistic Target Of Rapamycin) protein kinase, from an immunosuppressant to the most reproducible pharmacological geroprotector in geroscience. This is a rapidly evolving field, with the number of basic science studies, clinical trials, and off-label use of mTOR inhibitors by the general public expanding quickly. We review findings in model organisms that have revealed potent benefits of rapamycin not only for longevity but for the function of multiple organ systems and on the hallmarks of aging. We review completed and ongoing clinical trials of rapamycin and analogs for diseases of aging in humans, and discuss the challenges and side-effects of rapamycin that may limit its translation from the laboratory to the clinic. While the jury is still out, we conclude that rapamycin—or molecules that similarly act to inhibit mTOR—may yet realize the century-old dream of extending healthspan and lifespan with a small molecule.

## Introduction

Calorie restriction (CR) without malnutrition remains the most robust non-genetic intervention that can maximize lifespan and healthspan in diverse species, from the humble budding yeast to worms, flies, rodents, and even non-human primates such as rhesus macaques [1, 2]. Importantly, CR not only extends lifespan, but it improves healthspan, protecting against multiple age-related diseases and conditions including Alzheimer’s disease, cancer, cardiovascular diseases, diabetes, and frailty in mice and non-human primates, and in humans CR promotes adaptations that protect against age-related disease [3–8].

The mechanisms by which CR promotes health and longevity remained poorly understood until this century, when techniques allowing targeted genetic inactivation of specific nutrient sensing pathways in model organisms enabled systematic investigation of the mechanisms behind CR. While many different pathways have been implicated, one of the most central pathways that has been implicated in the response to CR and the regulation of aging is the mechanistic (previously referred to as mammalian) target of rapamycin (mTOR). mTOR is a serine/threonine protein kinase that is inhibited by rapamycin, a compound produced by bacteria originally isolated from the soil of Easter Island that first attracted attention due to its ability to inhibit the proliferation of eukaryotic cells [9].

Since its initial discovery, a gathering tide of information regarding how rapamycin functions at the molecular level and its impact on cellular and organismal physiology has led to the startling realization that rapamycin can extend lifespan in many model organisms. Critically, rapamycin does not only extend years of life, but it appears to rejuvenate various organ systems and promote healthy aging in mice. Over the last few years, news about rapamycin has been released often, with many large new clinical trials announced within the time span of just a few months. Here, we review the evidence for mTOR as a regulator of aging, discuss the effects of rapamycin on the health and longevity of animal models, and discuss the evidence collected thus far on the safety and efficacy of rapamycin as a geroprotector in humans. Finally, we discuss ongoing and planned clinical trials of rapamycin for geroprotective purposes and diseases of aging, as well as the development of alternative strategies to inhibit mTOR.

## mTOR as a regulator of aging

mTOR signaling is evolutionarily conserved in diverse species, including mice and humans. The mTOR kinase serves as the catalytic core of two distinct protein complexes, mTOR complex 1 (mTORC1) and mTORC2, each of which is composed of shared as well as unique protein subunits and phosphorylate different substrates. mTORC1 is regulated by a wide range of nutrients and hormonal cues, most notably including amino acids [10]. mTORC1 activity drives anabolic processes through the phosphorylation of substrates that include ribosomal protein S6 kinase 1 (S6K1) and eukaryotic translation-initiation factor 4E-binding proteins (4E-BPs). mTORC1 activity also inhibits autophagy via phosphorylation of substrates including Unc-51-like autophagy-activating kinase (ULK1) [11]. In contrast to mTORC1, which is responsive to diverse and numerous environmental and nutrient cues, mTORC2 has primarily been characterized as an effector of phosphoinositide 3-kinase (PI3K) signaling; mTORC2 phosphorylates substrates including the kinase AKT, serum–glucocorticoid-regulated kinase (SGK) and protein kinase Cα (PKCα) in response to PI3K activity.

Over two decades ago, the first demonstration that mTOR signaling regulated aging was made with the discovery that genetic inhibition of mTORC1 signaling in *C. elegans* extends lifespan [12]. Studies in yeast and flies demonstrating similar effects of inhibiting mTORC1 signaling on lifespan soon followed [13–15]. A major advantage of studies in these model organisms as compared to rodents, especially at the time, was the ease of conducting genetic modifications, enabling insight to be gained into the mechanisms downstream of mTORC1 that might contribute to the benefits of reduced mTORC1 signaling on lifespan. These studies revealed that inhibiting S6K1 or translation-initiation factors also extended lifespan, and suggested that CR might indeed function in part by reducing mTORC1 signaling [13, 16, 17].

While studies in mammals naturally took longer, it was observed early on that mTORC1 signaling is lower in long-lived Ames dwarf mice than in wild-type controls [18]. Similar to results in lower organisms, studies have now found that genetic depletion of mTORC1 signaling via a variety of strategies extends lifespan and healthspan in mice [19–21]. Similar to the results found in model organisms, at least some of the benefits are likely mediated via reduced signaling through S6K1, as deletion of *S6K1* (*Rps6kb1*) extends lifespan [22, 23]. Other benefits may be mediated by other mTORC1 substrates; expression of dominant negative 4E-BP1 in specific tissues improves the metabolic health of mice [24, 25].

While studies in yeast and worms suggested that mTORC1 signaling is a key mediator of the response to CR, more recent studies in flies and mice suggest the true situation is more complicated. In flies, CR and inhibition of mTOR signaling have somewhat additive effects on lifespan, suggesting that CR and mTOR inhibition may work through overlapping but not identical mechanisms [26]. In mice, CR and rapamycin result in very different, non-overlapping molecular signatures [27–29]. In fact, CR-fed mice actually have elevated post-prandial mTORC1 activity relative to ad libitum-fed controls in some tissues [30]. That said, suppression of mTORC1 activity is required for at least some of the metabolic effects of CR in mice [30].

## Rapamycin extends lifespan

The compelling genetic evidence that mTORC1 signaling is an evolutionarily conserved regulator of healthy aging has naturally led to great interest in the pharmacological inhibition of mTOR signaling. Rapamycin is FDA-approved for use as an immunosuppressant following organ transplantation; it is also approved to treat specific symptoms related to some conditions associated with increased mTOR signaling such as tuberous sclerosis complex (TSC). As discussed below, use of rapamycin as a gerotherapeutic has been increasingly explored in clinical trials as well as off-label by the general public.

In contrast to the genetic interventions in mTOR signaling, which were first explored in model organisms, studies of rapamycin’s effect on lifespan were first conducted in mice, with the National Institute on Aging Interventions Testing Program (NIA ITP) showing in 2009 that rapamycin could extend the lifespan of genetically heterogeneous mice starting at 20 months of age [31]. Since that time, multiple studies of the effects of rapamycin on mouse lifespan have been performed; this includes multiple studies by the NIA ITP testing different doses and dosing regimens as well as independent studies by at least twelve other groups, making rapamycin the most reproducible geroprotective compound ever studied (Table 1). Studies have shown that rapamycin also extends lifespan in yeast, worms, and flies, demonstrating that the effects of rapamycin on lifespan are evolutionarily conserved [26, 32, 33].

**Table 1:** The effect of rapamycin on the lifespan of wild-type mice

Strain	Sex	Starting age	Rapa dose	Route	Control (days)	Δ lifespan (%)	References
**Wild-type mice—rapamycin alone via diet**							
UM-HET3	Female	20 months	14 ppm	Diet	~886	13	Harrison et al. 2009 [31]
UM-HET3	Male	20 months	14 ppm	Diet	~820	9	
UM-HET3	Female	9 months	14 ppm	Diet	843–891	18	Miller et al. 2011 [194]
UM-HET3	Male	9 months	14 ppm	Diet	780–851	10	
129/Sv	Female	2 months	1.5 mg/kg	SC 3x/wk 2 wks per 4	759	10	Anisimov et al. 2011 [195]
C57BL/6 J.Rj	Male	4, 13, 20 months	14 ppm	Diet	~ 900	~ 10^*a*^	Neff et al. 2013 [139]
C57BL/6 J.Nia	Male	19 months	14 ppm	Diet	954	−3.5^*NS*^	Zhang et al. 2014 [196]
C57BL/6 J.Nia	Female	19 months	14 ppm	Diet	874	5	
UM-HET3	Male	9 months	4.7 ppm	Diet	807	3^*NS*^	Miller et al. 2014 [197]
UM-HET3	Male	9 months	14 ppm	Diet	807	13	
UM-HET3	Male	9 months	42 ppm	Diet	807	23	
UM-HET3	Female	9 months	4.7 ppm	Diet	896	16	
UM-HET3	Female	9 months	14 ppm	Diet	896	21	
UM-HET3	Female	9 months	42 ppm	Diet	896	26	
C57BL/6 J.Nia	Male	4 months	14 ppm	Diet	806*	11*	Fok et al. 2014c,a, b [107]
C57BL/6 J.Nia	Female	4 months	14 ppm	Diet	826*	16*	
C57BL/6 J.Nia	Male	20–21 months	126 ppm for 90 d	Diet	914	14	Bitto et al. 2016 [36]
C57BL/6 J.Nia	Female	20–21 months	126 ppm for 90 d	Diet	960	9	
UM-HET3	Male	20 months	42 ppm	Diet	772	11	Strong et al. 2020 [198]
UM-HET3	Male	20 months	42 ppm for 3 mo	Diet	772	11	
UM-HET3	Male	20 months	42 ppm	Diet every other mo	772	9	
UM-HET3	Female	20 months	42 ppm	Diet	905	15	
UM-HET3	Female	20 months	42 ppm for 3 mo	Diet	905	4^*NS*^	
UM-HET3	Female	20 months	42 ppm	Diet every other mo	905	8	
C57BL/6 *Terc*^+*/*+^	Male	3 months	42 ppm	Diet	509	43	Ferrara-Romeo et al. 2020 [199]
C57BL/6 *Terc*^+*/*+^	Female	3 months	42 ppm	Diet	711	23	
UM-HET3	Male	Birth (via dam)	42 ppm until 45 days	Diet	783	11.9	Shindyapina et al. 2022 [35]
UM-HET3	Female	Birth (via dam)	42 ppm until 45 days	Diet	822	9.1^*NS*^	
C3B6F1	Male	6 months	42 ppm	Diet	718	28.6	Baghdadi et al. 2024 [200]
C3B6F1	Female	6 months	42 ppm	Diet	837	25.0	
C3B6F1	Male	6 months	42 ppm	Diet every other wk	718	18.4	
C3B6F1	Female	6 months	42 ppm	Diet every other wk	837	14.3	
C3B6F1	Male	6 months	42 ppm	Diet every other wk	716	16.6	Gkioni et al. 2025 [39]
C3B6F1	Female	6 months	42 ppm	Diet every other wk	815	17.4	
**Wild-type mice—rapamycin alone via I.P**							
C57BL/6 J.Nia	*MF*	22–24 months	4 mg/kg	IP every other day	~ 795	> 14^*a*^	Chen et al*.* 2009 [91]
C57BL/6 J.Nia	Female	20 months	2 mg/kg	IP 1x/5 days	897	7	Arriola Apelo et al. 2016a, b [34]
C57BL/6 J.Nia	Male	20–21 months	8 mg/kg for 90 d	IP 1x/d	925	14	Bitto et al. 2016 [36]
C57BL/6 J.Nia	Female	20–21 months	8 mg/kg for 90 d	IP 1x/d	847	0^*NS*^	
Mixed	Male	600–700 d	4 mg/kg	IP every other day	911	13	Fang et al. 2018 [201]
Mixed	Female	600–700 d	4 mg/kg	IP every other day	896	22	
CD1	Male	Day 4	10 mg/kg until 40 days	IP 1x/d	655	8.9	Aiello et al. 2022 [37]
CD1	Male	Day 30	10 mg/kg until 60 days	IP 1x/d	655	3.5^*NS*^	
CD1	Female	Day 4	10 mg/kg until 40 days	IP 1x/d	714	8.4	
CD1	Female	Day 30	10 mg/kg until 60 days	IP 1x/d	714	3.8^*NS*^	
**Wild-type mice—rapamycin via diet as part of a combination**							
UM-HET3	Male and Female	9 months	14 ppm with metformin	Diet	It’s complicated—see manuscript for details		Strong et al. 2016 [202]
UM-HET3	Male and Female	9 or 16 months	14 ppm with acarbose	Diet	It’s complicated—see manuscript for details		Strong et al. 2022 [40]
C3B6F1	Male	6 months	42 ppm with trametinib	Diet—rapa every other wk	716	27.4	Gkioni et al. 2025 [39]
C3B6F1	Female	6 months	42 ppm with trametinib	Diet—rapa every other wk	815	34.9	

The impact of rapamycin on median lifespan in mouse studies since 2009 where longevity or mortality rate was determined. Sex is listed separately for males and females where sex-specific data exists. The rapamycin dose listed for dietary administration indicates the drug concentration in the ad libitum fed diet; the dose listed for administration in water, or administered intraperitoneally (IP) or subcutaneous (SC) indicates the dose in mg per kg of body weight. Control indicates median lifespan of control group in days; Δ lifespan is the percent change in median lifespan (* indicates that mean is reported instead). *MF* indicates that the lifespan results were not broken down by sex or that sex was not reported; *a*: Lifespan study % increase was not determined; *NS*: Not Statistically Significant. Control lifespan and percentage change are estimated when precise information is not available from the authors or is not listed in the referenced study. Table is adapted from [172] *Nature Aging* [172] and used with permission

In addition, rapamycin is extremely robust to changes in conditions; rapamycin extends lifespan in multiple strains and in both sexes, although females typically benefit more than males under any given dosing regimen. Dosing regimens for rapamycin are very flexible, and beneficial effects of rapamycin on longevity have been observed even when rapamycin is dosed intermittently, when it is given for only a short period of time immediately postnatally, or when it is administered to older mice [34–37]. The robustness of rapamycin, as well as the fact that rapamycin and CR likely engage at least some overlapping molecular pathways, have led some to refer to rapamycin as a CR mimetic [38]. Finally, the effects of rapamycin are additive with other geroprotectors, including acarbose, metformin, and trametinib [39, 40].

Due to these impressive results, studies are now underway in dogs and in primates to discover if rapamycin can extend lifespan in these organisms. TRIAD (Test of Rapamycin In Aging Dogs) is a randomized trial of rapamycin (0.138–0.18 mg/kg rapamycin once per week or placebo) in healthy, middle-aged large (20 to 55 kg) dogs who are living as companion animals and who have been enrolled by their owners [41]. One hundred fifty-eight animals were enrolled as of 2025, and the study is anticipated to run for 3 years; no results are yet available, though preliminary studies have confirmed the feasibility of the approach [42]. In the primate study, a total of 66 middle-aged marmosets of both sexes (*Callithrix jacchus*) were dosed with either 1 mg/kg rapamycin or a control, with the lifespan and healthspan of the animals followed longitudinally. Although the results of this study have not yet been published, the preliminary results suggest that rapamycin-treated marmosets lived 15% longer than the control group [43].

## Rapamycin side effects and mTORC2

An important caution regarding the use of rapamycin—and something that has limited the clinical use of rapamycin for various human diseases as well as exploratory use as a gerotherapeutic—is side effects. In humans, the side effects of rapamycin include immunosuppression, hyperlipidemia, and hyperglycemia [44–47]. These effects can also be observed in rodents [19, 48, 49]; however, their potential impact on aging in mice is unclear, as mice housed in laboratory facilities are protected from many pathogens, they are relatively resistant to atherosclerosis, and glucose levels may not have the same relationship with longevity in mice and humans [50].

**Fig. 1:**
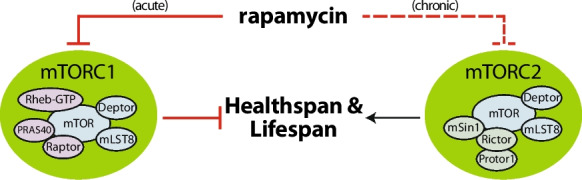
Differential roles of mTORC1 and mTORC2 in the regulation of healthy aging. Rapamycin acutely and potently inhibits mTORC1 while prolonged exposure to higher doses of rapamycin also inhibits mTORC2. As mTORC1 negatively impacts healthspan and lifespan, inhibition of mTORC1 by rapamycin is beneficial; but the inhibition of mTORC2 negatively impacts healthy aging. Adapted from Konopka *et al.* 2023, Geroscience [159] and used with permission

Rapamycin inhibits mTORC1 by first binding to FK506-binding protein 12 (FKBP12); this complex in turn then binds to the FKBP12-rapamycin-binding (FRB) domain of mTOR [51, 52]. While the FRB is located on the surface of mTORC1, in mTORC2 it is buried by proteins such as Rictor which are specific to mTORC2 [53–55]. Thus, while rapamycin does not acutely inhibit mTORC2, when high doses of rapamycin treatment are continued for a prolonged period of time, mTORC2 activity is decreased, most likely due to rapamycin sequestering free mTOR and hindering the formation of new mTORC2 [56]. This can be seen in most mouse tissues as well as in human cells in culture, where as outlined in Fig. 1, acute treatment with rapamycin inhibits mTORC1, while chronic treatment with rapamycin inhibits both complexes [19, 56, 57].

Interestingly, the metabolic side effects of rapamycin are mediated not by inhibition of mTORC1, but through off-target inhibition of mTORC2. This can be seen most clearly in the control of blood glucose; the negative impact of rapamycin on hepatic insulin resistance, leading to glucose intolerance, is the result of inhibition of mTORC2 in the liver [19]. Inhibition of mTORC2 also leads to insulin resistance in tissues including adipose, muscle, and the brain, disrupts lipid homeostasis, and negatively impacts both survival and frailty in mice [58–62]. Similar results are observed in model organisms, with genetic inhibition of mTORC2 activity in the heart impairing cardiac function in flies, while genetically increasing mTORC2 preserves cardiac function with age and extends the lifespan of flies [63, 64]; inhibition of mTORC2 also negatively impacts survival in *C. elegans* [65, 66]. Finally, the immunosuppressive effects of rapamycin depend at least in part on its suppression of mTORC2 activity [60, 67].

Conveniently from the perspective of developing gerotherapies, while mTORC2 inhibition seems to largely be negative with respect to lifespan, healthspan, and metabolism, inhibition of mTORC1 or signaling downstream of mTORC1 has been shown to be sufficient to extend lifespan in worms, flies, and mice [14, 16, 19, 21, 22, 32]. Thus, we hypothesized that treatments that expose subjects to rapamycin intermittently, especially at low doses, are likely to inhibit mTORC1 while largely not inhibiting mTORC2, and have reduced side effects while still extending lifespan, and we have shown that this is true in mice [34, 68]. As discussed below, this intermittent dosing strategy has proven popular in clinical trials of rapamycin for geroprotection; the results of these trials also suggest that mTORC1-specific inhibitors may enable the potential benefits of rapamycin to be harnessed with fewer side effects. While intermittent therapy seems likely to be safer than daily treatment, it also leads to less mTORC1 inhibition, and thus potentially could be less efficacious in its ability to promote healthy aging.

## Rapamycin (probably) extends healthspan

There are many positive effects of rapamycin on healthspan that have been characterized, primarily in mice. As highlighted in Fig. 1, these studies demonstrate improvements in aspects of health and function of many different organ systems, including the brain, the heart, the immune system, the intestine, the liver, the skin, skeletal muscle, joints, and tendons.

### Brain

Higher brain mTORC1 activity is associated with Alzheimer’s disease (AD) in humans [69–71] and is elevated in normal mouse aging as well as mouse models of AD [6, 72]. For this reason, rapamycin has been explored for its potential not only to fight age-associated cognitive decline, but also prevent or delay AD in mice. Rapamycin has been shown to block the development of AD pathology and cognitive decline in multiple mouse models of the disease [73–77], and to ameliorate the effects of aging on learning in wild-type mice as well [78]. Rapamycin also slows brain aging as assessed by DNA methylation clock [79]. While the mechanisms behind the beneficial effects of rapamycin for AD are unknown, several studies suggest that the benefits may be due to the induction of autophagy [76, 80] or the inhibition of S6K1 [81].

Initial studies in humans using a low dose of rapamycin (1 mg/day) were not successful in altering AD biomarkers in individuals with mild cognitive impairment, likely due to the lack of detectable rapamycin in the cerebrospinal fluid [82], but trials of the same low dose in cognitively normal APOE4 carriers may have successfully countered early vascular and metabolic deficits of the disease [83].

### Heart

Two studies in mice have examined the effect of rapamycin on age-related diastolic dysfunction, a condition analogous to heart failure with preserved ejection fraction (HFpEF), a common, debilitating, and lethal condition. These studies found that late-life, short-term treatment with rapamycin reversed diastolic dysfunction and cardiac hypertrophy, and attenuated age-related oxidative damage and rejuvenated the cardiac proteome [84, 85]. Further, these changes persisted for months following the cessation of treatment, as did increases in the expression of electron transport chain complex components [86]. Ex vivo studies found that rapamycin normalized age-related impairments in cardiomyocyte relaxation, as well as reducing myocardial stiffness [87].

These studies strongly suggest that rapamycin could be beneficial for a number of different age-related cardiac disorders. Rapamycin was recently shown to have benefits for feline hypertrophic cardiomyopathy in cats in a randomized control trial [88], and a delayed release formulation of rapamycin has now received conditional FDA approval for the treatment of subclinical hypertrophic cardiomyopathy in cats. A small study suggests that rapamycin also results in improvements in measures of both diastolic and systolic cardiac function in aging dogs [89]. There is substantial interest in trying rapamycin for the treatment of HFpEF, a disorder which substantially limits activity and decreases quality of life for millions in the United States alone and for which there is no satisfactory treatment. Initial pilot studies suggest that in older male subjects without cardiac disease, rapamycin (1 mg/day) can improve multiple measures of cardiac function, including blood flow, flow rate, and the acceleration of blood as it exits the left ventricle [90].

### Immune system

mTOR inhibition with rapamycin improves the function of the aging immune system in both mice and humans. A 6-week course of rapamycin rejuvenates the function of hematopoietic stem cells (HSCs), increases production of naive lymphocytes, and improves the response to influenza vaccination in old mice [91]. An important caveat, however, is that rapamycin, even when given at low doses or intermittently, is an immunosuppressant, though intermittent rapamycin treatment may be less immunosuppressive due to reduced inhibition of mTORC2 [67, 68, 92–94].

In humans, 6 weeks of treatment with an mTOR inhibitor (everolimus or a catalytic site mTOR inhibitor) likewise improved the response to influenza vaccination, and was further associated with a decrease in the percentage of exhausted PD-1^+^ T cells in peripheral blood [95, 96]. mTOR inhibition has also been shown to upregulate antiviral immunity in older adults [96, 97]. Ex vivo analysis of aged immune cells from older adults found evidence of DNA damage and mTOR hyperactivation, and rapamycin treatment (1 mg/day for 4 months) of humans reduced levels of p21, a DNA damage and senescence marker [98]. While there is a general thought that intermittent treatment with rapamycin may be less immunosuppressive due to the decreased impact on mTORC2, self-reported infections of many different types among individuals self-administering rapamycin for geroprotection trended higher [99], and a physician prescribing rapamycin for geroprotection informed his patients that they were at an increased risk of bacterial infections [100].

### Intestine

Rapamycin increases the self-renewal capacity of mouse intestinal stem cells (ISCs) via a non-cell-autonomous mechanism mediated by inhibition of mTORC1 in the adjacent Paneth cells [101]; similar effects of rapamycin have been observed in human cells [102]. Inhibition of mTORC1 in quiescent mouse ISCs also protects against DNA damage [103]. This may proceed by counteracting an age-associated activation of mTORC1 in mouse intestinal stem and progenitor cells, which drives activation of p38 MAPK-p53 signaling [104]. In flies and mice, the benefits of rapamycin for age-related gut pathology are mediated in a sexually dimorphic manner by increased autophagy, with greater benefits for females than males [105].

### Liver

Rapamycin has been shown to reduce the accumulation of age-associated pathology in multiple tissues of mice, including the liver [106]. Further, rapamycin treatment rejuvenates the liver transcriptomic profile of aged mice [107] and reduces the epigenetic age of mouse liver as assessed by DNA methylation [108, 109]. While the mechanism for these benefits is unknown, autophagy is impaired with age in multiple tissues of mice, and genetic activation of autophagy in the aged mouse liver rejuvenates the liver histologically and improves its function [110]. While rapamycin is less effective at inducing autophagy than mTOR kinase inhibitors [111], multiple studies report that rapamycin can induce autophagy *in vivo* [76, 105].

There are also a number of studies that demonstrate the ability of rapamycin to protect mouse livers against ischemia; these effects have been linked to the induction of autophagy as well as the activation of mTORC2 [112, 113]. Rapamycin also increases resistance to ischemia in ex vivo perfused livers, reducing necrosis despite only a brief 3 h treatment with rapamycin [114].

### Skin

While we are all familiar with the changes in skin appearance with age, the skin also undergoes many age-associated changes that compromise its function [115]. Although systemic rapamycin use is associated with an increase in bacterial and fungal infections of the skin and nails, as well as dermatitis [116], topical treatment of subjects in a recent clinical trial did not result in detectable levels of rapamycin in the subjects’ blood, and there were no treatment-related adverse events [117]. In this trial, topical administration of rapamycin was shown to improve skin appearance, reduce the expression of the cellular senescence marker p16, and increase collagen VII protein expression [117]. Rapamycin also had a favorable effect on skin lesions in a mouse model of AD [118]. While rapamycin is not clinically approved for the treatment of age-related skin conditions, RLX-201, a rapamycin derivative that has been shown to selectively reduce mTORC1 activity in skin fibroblasts [119], is now being offered to the public as part of a physician-dispensed skincare regimen [120].

### Musculoskeletal system

There has been substantial interest in using rapamycin to improve components of the musculoskeletal system, the deterioration in function of which is a major cause of disability. Higher mTORC1 and/or S6K activity has been observed in the muscle of older mice, rats, and humans, and thus is associated with sarcopenia [121–125]. In a beautiful study, it was shown that late-life treatment with a low dose of rapamycin for just 6 weeks not only suppressed this hyperactive mTOR signaling, and reversed sarcopenia in rats, actually increased the mass of some muscles, while halting the age-associated loss of mass in others [123]. Molecular changes associated with sarcopenia were also partially reversed [123].

These results sound very promising, and although genetic inactivation of mTORC1 in skeletal muscle in adult mice has been shown to have some negative effects on muscle function [126], other studies have shown that mTORC1 function is dispensable in adult mice for the maintenance of muscle mass [127]. Indeed, genetic inactivation of 4E-BPs has been shown to protect mice from age-associated sarcopenia [128]. As rapamycin can compromise the function of mitochondria in cultured muscle cells, there has been some concern that rapamycin could compromise muscle strength or interfere with the benefits of exercise on muscle; however, studies in mice thus far suggest these concerns are unfounded [129, 130]. However, rapamycin has been shown to inhibit mitochondrial complex I activity in guinea pig muscle [131]. In some preliminary studies, a small randomized clinical trial of rapamycin in humans, PEARL, reported that intermittent rapamycin treatment (10 mg of compound rapamycin/week), which should inhibit mTORC1 while leaving mTORC2 signaling largely intact, led to increased lean mass in women [132].

There has been less, but still significant research on the effect of rapamycin on other components of the musculoskeletal system. Rapamycin treatment prevents the age-related increase in tendon stiffness in mice, as well as blocking age-associated calcification of the Achilles tendons [133]. However, the effects of rapamycin on osteoarthritis appears more mixed; in guinea pigs, rapamycin treatment was associated with greater osteoarthritis severity due to articular cartilage structural damage and/or proteoglycan loss that correlated with rapamycin-induced hyperglycemia [134]. Follow-up studies suggest that rapamycin results in deleterious effects on osteoarthritis pathology in both mice and marmosets due to feedback activation of Akt signaling [135]. While this is definitely concerning from the standpoint of systemic administration of rapamycin as a gerotherapy, several studies in mice and guinea pigs suggest that intra-articular administration of rapamycin, alone or in combination with other therapies, may have benefits for the treatment of osteoarthritis [136–138].

### Healthspan

As the above makes clear, studies in mice as well as limited studies in cats, dogs, non-human primates, and humans all suggest that rapamycin can blunt or reverse age-related declines in function in multiple organ systems. One might then be tempted to jump to the conclusion, as many do, that rapamycin extends not only lifespan, but also healthspan. While the accumulating evidence certainly points in that direction, there are reasons to be cautious in reaching such a conclusion. For example, while a study of the effects of rapamycin on certain specific healthspan markers in HET3 mice came to the conclusion that rapamycin improves healthspan [106], an extensive analysis of healthspan markers in C57BL/6 J males concluded that the main effect of rapamycin was on cancer, and that healthspan and the “rate of aging” was unaffected [139].

While healthspan is relatively easy to define—e.g., “the period of life spent in good health, free from the chronic diseases and disabilities of aging” [140], quantifying healthspan is trickier. We have proposed two measurements analogous to quality adjusted life years (QALY), the gold standard for measuring the effects, positive or negative, of medical treatments on patients’ lives [141]. These measurements, FAMY and GRAIL, integrate lifespan data with either longitudinal frailty data (FAMY) or longitudinal frailty data with information about the hallmarks of aging from physiological and molecular measurements (GRAIL) [5]. Other proposed approaches include scoring healthspan as the period of life in which mice exhibit fewer than four severe deficits [142]. Irrespective of the details, these approaches all require longitudinal data on frailty collected on animals engaged in a lifespan study; and perhaps surprisingly, this has not yet been done for rapamycin. The one study that measured frailty in a cross-sectional cohort reported that rapamycin reduces frailty; however, in this study, mice were only treated with rapamycin during early life [35].

Alternative but related concepts include those of intrinsic capacity and resilience; and certainly, the data presented above might lead one to think that rapamycin increases both intrinsic capacity and resilience. However, as has been found with calorie restriction—which indisputably increases both lifespan and healthspan in mice [5, 59, 143]—the effects of rapamycin on intrinsic capacity and resilience is less clear-cut. Rapamycin-treated mice tend to be lean [34, 144, 145], which while healthier means they likely have decreased resilience to fasting. Rapamycin-treated mice also have decreased cold tolerance, and when challenged with cold their core temperature drops more and they lose more weight than vehicle-treated controls [146]. Rapamycin is well documented to impair wound healing in multiple species including mice [147–151], and also impairs the early healing of bone fractures in mice [152]. Of course, rapamycin is also an immunosuppressant, and multiple studies in mice show that rapamycin impairs the response to acute infections as well as adaptive immunity in mice [93, 94]; many studies show that humans subject to rapamycin and rapalogs have an increased risk of infection, particularly bacterial infections that can lead to hospitalization and death [153].

## Rapamycin in humans

### Completed clinical trials of rapamycin for geroprotection

As discussed briefly above, some of the most compelling clinical applications of rapamycin have focused on reversing aspects of age-related immune dysfunction. Dr. Joan Mannick and colleagues evaluated three different dosing regimens of the rapalog everolimus in subjects aged 65 years and older [95]. In this randomized, placebo-controlled phase 2 trial, subjects received low-dose everolimus (0.5 mg) daily or received a higher dose of everolimus weekly (5 mg or 20 mg) for 6 weeks; after a 2-week drug washout, the subjects then were vaccinated against influenza. The study found that 0.5 mg everolimus once daily or 5 mg everolimus once weekly was well tolerated by the subjects, and importantly significantly improved the response to influenza vaccination.

These findings were extended in a subsequent randomized clinical trial examining treatment with either everolimus, the catalytic mTOR inhibitor BEZ235, or a combination in subjects aged 65 years and older [96]. This study also found that 6 weeks of treatment with mTOR inhibitors was safe, and importantly observed that the treatment regimen not only improved the influenza vaccination response, but also decreased the rate of total infections. Transcriptional profiling of blood cells found that the older adults receiving mTOR inhibitors at low doses had significant upregulation of genes in antiviral pathways, which may underlie the decreased incidence of respiratory tract infections. The overall safety and benefits of low-dose mTOR inhibitors for the immune system were confirmed in studies that treated older adults for 16 weeks. Low doses of the mTOR inhibitor BEZ235 were well tolerated and decreased the incidence of laboratory-confirmed RTIs, but did not reduce the incidence of clinically symptomatic respiratory illness [97].

A separate randomized controlled trial evaluated daily rapamycin administration (1 mg/day) in healthy older adults for 8 weeks [154]. While rapamycin treatment in this study was generally well tolerated, some metabolic side effects were observed, including small increases in glycated hemoglobin (A1C) (within-group *p* = 0.03) and a 40% increase in triglyceride levels (within-group *p* = 0.05). Although these results align with mouse and human studies suggesting that chronic rapamycin treatment can cause hyperglycemia and hyperlipidemia, this was a very small study, and the between-group comparisons with a placebo control group did not reach statistical significance (*p* = 0.07 and 0.12 for A1C and triglyceride changes, respectively).

As discussed briefly above, rapamycin has also been tested on skin; an important advantage of topical administration is that topical administration resulted in undetectable levels of rapamycin in the blood, and subjects experienced no treatment-related adverse events. In this randomized placebo-controlled study of participants aged ≥ 40 years with signs of skin aging, 36 participants topically applied cream containing rapamycin (10 μM) to one hand and a matching placebo cream to their other hand for 8 months [117]. Rapamycin reduced the number of senescent cells as assessed by p16 expression, increased the expression of collagen VII protein, and was found to improve skin appearance.

The Participatory Evaluation of Aging with Rapamycin for Longevity Study (PEARL), led by Dr. Sajad Zalzala of AgelessRx, recently reported results from a 48-week long decentralized, double-blinded, randomized, placebo-controlled trial [132]. Subjects took either placebo, 5 mg or 10 mg compounded rapamycin weekly; however, it was discovered that the compound rapamycin was less bioavailable than commercial formulations, and thus, the true doses were approximately 1.65 and 3.3 mg weekly [132]. So far, the results reported by PEARL indicate that these doses of rapamycin were well-tolerated, with similar levels of adverse events across groups, and a small increase in HbA1c for males on the lower dose of rapamycin. No effect of rapamycin was observed on the primary outcome, visceral adiposity. Lean tissue mass and self-reported pain improved significantly for women on the higher dose of rapamycin, while self-reported emotional well-being and general health improved for those using the lower dose of rapamycin.

A phase 1 trial of daily (1 mg/day) rapamycin was recently conducted over the course of 8 weeks with 10 adults 55—85 years old with AD or mild cognitive impairment. Somewhat surprisingly, the primary outcome, rapamycin penetrance, showed that rapamycin was not detectable in the cerebrospinal fluid (CSF) [82]. There were a number of adverse events, including two mild ones (urinary urgency and diarrhea) possibly related to the study, and there was a mild (3%) but statistically significant increase in hemoglobin A1c (HbA1c) and systolic blood pressure significantly increased by 8%. Plasma levels of Aβ40 and Aβ42 decreased (*p* = 0.137 and *p* = 0.07, respectively), while CSF levels of pTau 181, GFAP (glial fibrillary acidic protein), and NfL (neurofilament light) all significantly increased [82]. The poor brain penetrance of rapamycin has also been observed in other studies, including a randomized, double-blind trial of rapamycin for amyotrophic lateral sclerosis where the primary endpoint was not attained and rapamycin was not detected in the CSF [155]. These results are surprising, as rapamycin is efficacious at treating cerebral manifestations of tuberous sclerosis complex (TSC) and other mTORopathies; however, rapamycin in TSC is often used at much higher doses [153], and those doses used here may be too low to be detectable in CSF. The paradoxical increase in plasma Aβ40 and Aβ42 could reflect an outflux of materials from brain plaques that are now being removed from the brain.

Finally, a recent study investigated the potential interaction between exercise and rapamycin; the investigators hypothesized that rapamycin would enhance adaptation to exercise. The randomized, double-blinded trial assigned 40 sedentary adults to either placebo or 6 mg of rapamycin once weekly for 13 weeks [156]. In contrast to the investigator’s hypothesis, rapamycin impaired the response to exercise, with subjects taking rapamycin able to do significantly fewer chair-stand repetitions than those taking placebo. There was also a trend towards rapamycin impairing grip strength and reducing quality of life scores, and one subject taking rapamycin had a serious adverse event, pneumonia.

In total, these studies suggest that rapamycin may have potential benefits for healthy aging, with initial studies suggesting that rapamycin may be able to rejuvenate the skin and the immune system, reducing senescent cells in the skin, improve the response of older adults to vaccination, and potentially bolster lean mass, reduce pain, and improve overall health. These studies also suggest that when rapamycin is used intermittently or topically, and doses are low, leading to minimal mTORC2 inhibition, rapamycin has an acceptable side-effect profile. However, these studies are typically quite small and conducted for only a limited time; thus, the long-term benefits, if any, as well as the side-effects of rapamycin treatment remain to be determined. The interaction of rapamycin with other interventions to promote health, including exercise, needs continued study, as an initial study suggests that rapamycin use may result in reduced health gains from exercise. There remains a critical need for studies that follow larger number of people for longer periods of time, both to determine the side effects of rapamycin and to comprehensively assess the effects of rapamycin on the hallmarks of aging and intrinsic capacity.

### Off-label use of rapamycin

The many positive effects of rapamycin in animal models have spurred significant interest in the use of rapamycin by longevity enthusiasts including clinicians, scientists, and the public. While no one can stop physicians from prescribing rapalogs to the public in order to fight aging—rapamycin is FDA-approved and thus off-label use is legal in the U.S.—in addition to the risk of immunosuppression, rapamycin use is associated with hyperlipidemia, hyperglycemia, and an increased risk of new onset diabetes [44, 154]. In addition to several clinical trials of rapamycin and everolimus that are underway or have been performed, it is estimated that at least 18,000 people are taking rapamycin “off-label” under the care of private physicians in New York, LA, and elsewhere, and new rapamycin telemedicine services have now launched [157].

This population is potentially a unique resource for studying the real-world effects of rapamycin. A study of this population, comparing 333 rapamycin users to 172 non-rapamycin users as controls, reported that most of this population used rapamycin intermittently, following a once a week dosing schedule, across a range of doses. While this study was performed on a self-selected population—individuals who experienced serious side effects likely stopped taking rapamycin and did not participate in the study—users did not report many adverse events; mouth ulceration was the only self-reported adverse event that was greater in rapamycin vs. non-rapamycin users [99]. The users were not blinded, and thus, reports of the effects of rapamycin on perceived health are subject to the placebo effect; nevertheless, rapamycin users believed that rapamycin improved, with ~ 25–38% of rapamycin users reporting that they felt younger, more confident, and more energetic, or reporting that rapamycin had other perceived health benefits. However, ~ 62–75% of rapamycin users did not perceive these benefits. As no clinical lab results were evaluated, and users were assessed at only a single point in time, these results should be interpreted cautiously.

Many of the subjects assessed in this study were patients of Dr. Alan Green, a geriatrician in New York City, who began self-administering rapamycin at a dose of 6 mg/1 × week to treat his own hypertrophic cardiomyopathy in 2016, and began prescribing it to his patients as a geroprotector the following year [100]. Dr. Green believed that rapamycin cured his hypertrophic cardiomyopathy, and also stated “that weekly rapamycin had not only improved my heart condition; but also my prostate symptoms, periodontal gingivitis, overweight and a general feeling of being old” [100]. Based on his observations of his ~ 1500 patients, Dr. Green believed weekly rapamycin increased the risk of bacterial infections roughly twofold, and he stressed the importance of aggressively treating bacterial infections and stopping rapamycin if you had an infection; however, he believed that weekly rapamycin generally did not have other clinically significant side effects [100]. Side effects of self-administered rapamycin have also been reported by Bryan Johnson, a tech entrepreneur who has gained significant attention for his longevity regimen and who reported in 2025 that he had ceased taking rapamycin due to its side effects. He reported that while taking various doses of rapamycin on once weekly or alternating weekly schedules, he experienced intermittent skin/soft tissue infections, lipid abnormalities, glucose elevations, and an increase in his resting heart rate [158].

### Ongoing clinical trials of rapamycin for geroprotection

To gain more insight not just into the safety of rapamycin but its potential use as a gerotherapeutic, several studies are now underway that propose to examine the impact of rapalogs on diseases of aging (Table 2). We have previously provided a detailed description of the rationales, subjects, and primary and secondary outcomes for these studies [159], and to the best of our knowledge all of these studies are now underway.

**Table 2:** Summary of ongoing or upcoming rapamycin clinical trials. Table adapted from [159] Geroscience [159] and used with permission

Population	mTOR inhibitor dosing regimen	Duration	Study design	Primary endpoint	Reference
10 young men (22 − 35y)	Single dose of rapamycin 16 mg or placebo	Once	Non-randomized, double-blinded, placebo controlled, cross-over design	Post-exercise, insulin stimulated muscle glucose uptake	NCT05233722, Communication with Jørgen Wojtaszewski
72 adults 55—80y	Rapamycin 5, 10, or 15 mg weekly Everolimus 5, 10, or 15 mg weekly	6 weeks	Allocated, open label, dose finding trial	Dose limited toxicities to determine RP2D	RAP PAC, NCT05949658, Communication with Adam Konopka
50 patients with periodontitis	Rapamycin 5 mg weekly Placebo	8 weeks	Randomized, placebo-controlled	Clinical attachment loss	Communication with Jonathan An
50 Women with premature ovarian failure	Rapamycin 5 mg weekly Placebo	12 weeks	Randomized, placebo-controlled	Ovarian reserve	VIBRANT, NCT05836025 Communication with Samuel Williams and Yousin Suh
194 adults 65—90y (84 for final study)	Rapamycin, dose finding, daily and/or weekly Everolimus, dose finding, daily and/or weekly	12 weeks and 12 months	Randomized, Open label (initial study), double-blinded (final study)	Pharmacology of each drug, p-S6 in blood cells, and serum sICAM-1	RESTOR, NCT06658093, [203]
60 adults 65—80y	Rapamycin 0.5, 1, or 2 mg daily Everolimus 0.5, 1, or 2 mg daily	3 months	Randomized, open label, dose adjusted trial	Pharmacology of each drug, SASP, laboratory and function biomarkers of aging	NCT06727305
16 males age ≥ 65y	Rapamycin 1 mg daily Placebo	16 weeks	Randomized, single-blinded, Placebo-controlled, unilateral resistance exercise training vs. sedentary contralateral leg	Change in muscle mass	NCT05414292
72 adults with insulin resistance (55—80y)	Everolimus 0.5 mg daily Everolimus 5 mg weekly Placebo	24 weeks	Randomized, double-blinded, placebo-controlled	Change in peripheral insulin sensitivity	EVERLAST, NCT05835999, Communication with Adam Konopka
15 patients with early AD	Rapamycin 7 mg weekly	6 months	Open Label	Change in cerebral glucose metabolism	NCT04629495
22 adults (65—95y)	Rapamycin 8% ointment topically applied daily Placebo	6 months	Randomized, double-blinded, Placebo-controlled,	Change in epigenetic markers	Topical-RAPA, NCT04608448
40 patients with MCI/early AD	Rapamycin 1 mg daily Placebo	12 months	Randomized, double-blinded, placebo-controlled	Number of adverse events	REACH, NCT04629495, Communication with Mitzi Gonzales
726 adults (60—65y)	Rapamycin daily Placebo Dapagliflozin Semaglutide	12 + months	Randomized, double-blinded, placebo-controlled	Intrinsic Capacity	[161–163]
Adults 65 and older	Rapamycin Placebo	3 years	Randomized, double-blinded, placebo-controlled	Physical function and IL-6	[164]

A study we did not previously discuss is Topical-RAPA Use in Inflammation Reversal and Re-setting the Epigenetic Clock (Topical-RAPA), NCT04608448. This randomized, double-blinded placebo-controlled trial is examining the effect of rapamycin on epigenetic aging of the skin. Participants between 65 and 95 years of age applied a lotion containing 8% rapamycin to one forearm daily, and a placebo lotion to the other arm; thus, each subject serves as their own control. Skin epigenetic age and blood inflammatory markers were measured at baseline and after 6 months. Twenty subjects completed the trial as of 2023, but the results of this study have not yet been published.

A new study we have not previously discussed is the Characterization of mTOR Inhibitor Pharmacokinetics and Pharmacodynamics in Older Adults study (NCT06727305). This randomized, open-label trial will test mTOR inhibitors in 60 older adults between 65 and 80 years of age. Participants will receive either rapamycin or everolimus at a dose of either 0.5 mg, 1 mg, or 2 mg per day for 2 weeks, and then following pharmacokinetics and pharmacodynamics testing, dose adjustments will be made to obtain stable blood levels of 5–7 ng/ml for 3 months. Most of the primary outcomes relate to the pharmacokinetics and pharmacodynamics of the two compounds tested, but at the completion of the study cellular senescence will be assessed through measurements of the SASP, and laboratory assays of aging will be assessed, including the activity of S6K, levels of C-reactive protein, and function will be assessed through a 6-min walk test and a standardized short physical performance battery to assess functional mobility.

Some of these studies have attracted significant interest from the press, including the VIBRANT (Validating Benefits of Rapamycin for Reproductive Aging Treatment) trial for rapamycin in ovarian aging. A pilot trial in 50 healthy women of reproductive age receiving rapamycin weekly for 3 months has been successfully completed according to a recent report, with subjects tolerating the treatment and no significant adverse events [160]. A follow-up trial (VIBRANT II) with 200 subjects is reportedly planned on the basis of these findings [160], but as of this writing no publications have been forthcoming.

A newly announced trial is RESTOR: PK/PD mTORi Inhibition in Older Adults (NCT06658093). This is a large, multi-aim study that will enroll up to a total of 194 subjects, including a young control group (20–30 years of age) and an older study group (65–90 years of age). Aim 1 of RESTOR will be an open-label dose finding study, testing a range of doses of rapamycin and everolimus given either daily or intermittently; the primary outcomes will be pharmacokinetics and pharmacodynamics and the level of Soluble Intercellular Adhesion Molecule-1 (sICAM-1). Aim 2 of RESTOR will be a randomized, double-blinded trial conducted in 84 aged subjects, which will test the optimized daily and intermittent dosing regimens over 12 months (6 months of treatment with a 6-month follow-up) identified in Aim 1; pharmacokinetics and pharmacodynamics will be the primary outcome of this Aim as well.

Two additional rapamycin trials have recently been announced. The PROactive Solutions for Prolonging Resilience (PROSPR) program of the Advanced Research Projects Agency for Health has announced funding for a phase 3 hybrid trial of rapamycin and two other potential gerotherapeutics by the University of Texas Health Science Center at San Antonio [161]. This $38 million randomized, double-blind trial will enroll a total of 726 adults between the ages of 60 and 65, all of whom will take one pill daily, and follow them for at least a year to assess the effects on intrinsic capacity [162, 163]. Finally, the University of Arizona has announced a $12 million study which will conduct a randomized, double-blind trial of rapamycin in adults 65 years of age and older for three years (2 years of treatment and 1-year follow-up). The primary outcomes of this study will be physical function and the transition to frailty, as well as inflammation as assessed by blood levels of IL-6 [164].

## Novel mTOR inhibition strategies

While researchers continue to explore the clinical use of rapamycin as a gerotherapeutic, there is strong interest in identifying new ways to selectively inhibit mTORC1 without off-target inhibition of mTORC2, which could lead to potent therapies that have fewer side effects than rapamycin. A number of dietary interventions, including ketogenic diets and diets with reduced levels of either protein or specific amino acids have been shown to reduce mTORC1 activity and extend lifespan in mice [165–171]. Importantly, mice fed these pro-longevity diets do not suffer from the metabolic and immune side-effects commonly seen with rapamycin, providing further support for the idea that it should be possible to inhibit mTORC1 without these negative consequences.

**Fig. 2:**
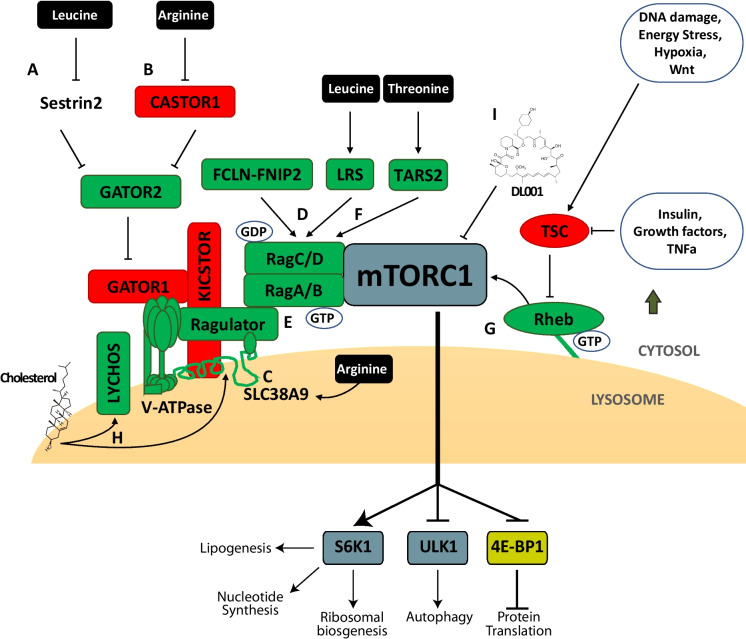
An overview of canonical mTORC1 activation at the lysosome with areas of potential pharmaceutical inhibition highlighted. Negative regulators (CASTOR1, GATOR1, SAMTOR, sestrin 2, TSC) and positive regulators (FLCN–FNIP2, GATOR2, KICKSTOR, LRS, Rag GTPases, Ragulator, RHEB, SLC38A9, vacuolar-type ATPase (V-ATPase)) are shown. Potential mechanisms for the development of mTORC1-specific inhibitors include: identifying small molecules that block the ability of amino acid sensors upstream of mTORC1 to sense the availability of leucine or arginine or SAM (**A**–**C**); developing compounds such as BC-LI-0186 that inhibit the GAP or GEF activities of FLCN–FNIP2, LRS, Ragulator or TARS2 (**D–F**); inhibiting the interaction of mTORC1 and RHEB, the mechanism of action of NR1 (**G**); identifying small molecules that block the ability of LYCHOS and SLC38A9 to sense the availability of cholesterol (**H**); and identifying rapamycin derivatives such as DL001 that specifically inhibit mTORC1 (**I**). TNF, tumor necrosis factor-α. Select downstream substrates of mTORC1 and processes mediated by them are also shown. Figure is adapted from Mannick and Lamming 2023, *Nature Aging* [172] and used with permission

The molecular regulation of mTORC1 by amino acids and other nutrient and environmental cues by numerous distinct protein complexes at the lysosomal surface has been investigated in detail over the past two decades, and is reviewed in-depth elsewhere [172, 173]. We highlight the major proteins, protein complexes, and molecules involved in the now-canonical regulation of mTORC1 activity in Fig. 2. Briefly, mTORC1 is recruited to the lysosomal surface by interacting with heterodimeric pairs of the Rag family of small GTPases [174, 175]; amino acids regulate mTORC1 by controlling the GTP binding state of the Rags [176–179].

The significant advances in understanding the molecular regulation of mTORC1 and the structure of its associated regulators has provided insight on potential new ways (highlighted in Fig. 2) to specifically target mTORC1, which could lead to compounds that avoid off-target inhibition of mTORC2. Compounds in the class include (*S*)−4-isobutyloxazolidin-2-one and BC-LI-0186, which inhibit mTORC1 by interfering with Leucyl-tRNA Synthetase (LRS) sensing or activity towards RagD [180–184]. Many other potential sites on other amino acid or cholesterol sensors that signal nutrient availability to mTORC1 could potentially be targeted by future compounds.

Depending on the exact mechanism, some new ways of inhibiting mTORC1 may inhibit mTORC1 activity more broadly than rapamycin, which inhibits only a subset of mTORC1 activity [111]. This could be beneficial for lifespan and healthy aging, as such compounds could more effectively activate beneficial processes like autophagy, or be detrimental by interfering with processes that are normally not altered by rapamycin [111]. Examples of these types of compounds include NR1, which binds the mTORC1 activator RHEB and prevents it from allosterically activating mTORC1, and acts as a specific inhibitor of mTORC1 [185]. Finally, Rapalink-1, a compound in which an mTOR kinase inhibitor is linked to rapamycin and delivered at a low dose, selectively inhibits mTORC1 kinase activity, and has shown efficacy in animal models [186, 187]. RMC-5552, a similar “bi-steric inhibitor” is being developed for cancer treatment and is currently in clinical trials [188, 189].

Another possible route to the further development of mTORC1 inhibitors is the development of mTORC1-selective rapalogs [190]. We reported in 2019 the identification of DL001, a compound with significantly greater selectivity for mTORC1 than rapamycin; mice treated with this compound had substantially reduced glucose intolerance, dyslipidemia, and immune disruption as compared to mice treated in parallel with rapamycin [60]. Other companies have also announced their intent to develop mTORC1-selective rapalogs [191]. One such compound, NV-20494, has shown efficacy in a mouse model of polycystic kidney disease and in vitro in human three-dimensional cell culture [192], and prolongs the lifespan of progeroid *Lmna*^H222P/H222P^ mice [193]. These types of molecules are moving closer to the clinic; Aeovian Pharmaceuticals recently announced the successful closing of a funding round to complete a phase 2 proof-of-concept study for AV078, a CNS-penetrant selective mTORC1 inhibitor, its lead development candidate, AV078, in TSC-related refractory epilepsy. This study follows the completion of a phase 1 study (NCT06205381) of this compound. The PROSPR program of ARPA-H has announced funding to support a study by Cambrian BioPharma to determine if a daily, oral, novel rapamycin analog will improve aging outcomes [161].

## Conclusions

Over the last two decades, rapamycin has moved from being simply an immunosuppressant to being one of the premier hopes of geroscience to not only treat, delay, or reverse numerous age-related diseases, but to extend healthy lifespan. Here, we have discussed the strong preclinical evidence—primarily from rodents, but increasingly from other mammalian models as well—that rapamycin can extend lifespan and healthspan. Clinical trials proving the efficacy of rapamycin for the treatment of age-related diseases in humans are still under way, and trials demonstrating the efficacy of rapamycin for extending healthy lifespan and slowing aging are for the most part just starting. With the important caveat that all the information is not yet in, initial short-term studies suggest that when dosed intermittently at low doses, rapamycin may be utilized under medical supervision with few adverse events.

As described here, the available evidence shows that rapamycin is a powerful intervention that engages with many of the hallmarks of aging. In multiple mammalian tissues, rapamycin attenuates dysregulated nutrient signaling, enhances proteostasis, counters cellular senescence and inflammation, promotes stem cell function, and promotes rejuvenation of immune function. This suggests that rapamycin has a broad capacity to modulate aging directly in a geroprotective fashion and does not simply treat the symptoms of disease. However, rapamycin can adversely impact at least a few hallmarks of aging, as long-term high dose rapamycin promotes deregulated nutrient sensing, and rapamycin is an acute immunosuppressant.

Outstanding and critical questions that remain to be answered include the longer-term safety of such regimens as well as their efficacy, as studies in mice generally suggest higher doses of rapamycin are more efficacious than lower doses. Studies in rodent models as well as marmosets suggest that rapamycin may potentially worsen osteoarthritis; as this could potentially be a substantially limiting side effect for longer-term treatment regimens, as the length of studies increase the potentially negative effect of rapamycin on joints must be carefully examined. While self-reported data from off-label use of rapamycin is broadly positive, this is a highly self-selected population, and even among these users there are reports from some, like Bryan Johnson, of negative metabolic impacts. Other outstanding questions include the brain penetrance of rapamycin, which may be a limiting issue, particularly for neurodegenerative disease, and drug sourcing, as the PEARL trial has found compounded rapamycin can be less bioavailable than rapamycin from the manufacturer.

It may be frustrating to many to wait for the results of randomized clinical trials, and some people may feel that they are not able to wait. We have no doubt off-label use of rapamycin or the dermatological use of mTOR inhibitors will continue despite outstanding questions regarding safety and efficacy. Ultimately, randomized clinical trials will be the venue in which the safety and efficacy of rapamycin and other mTOR-inhibitory therapies will be proven or disproven. These gold-standard answers will begin to arrive soon.
